# Follistatin like protein (FSTL-1) levels are elevated in acute Kawasaki Disease and may differentiate between patients with and without aneurysm formation

**DOI:** 10.1186/1546-0096-10-S1-A1

**Published:** 2012-07-13

**Authors:** Mark Gorelik, David Wilson, Stanford Shulman, Raphael Hirsch

**Affiliations:** 1 Childrens Hospital of Pittsburgh of UPMC, Pittsburgh, PA, USA; 2 Northwestern University, Chicago, IL, USA; 3 University of Pittsburgh, Pittsburgh, PA, USA

## Purpose

Kawasaki Disease is the most common vasculitis of childhood, and carries considerable morbidity with risk of development of coronary artery aneurysms. Serological predictors of the development of coronary artery aneurysms have not yet been found. FSTL-1 is a pro-inflammatory protein which is produced by mesenchymal tissue, including cardiac myocytes, and has been found to be elevated in inflammatory disorders such as Juvenile Idiopathic Arthritis. The current study was designed to determine if plasma levels of FSTL-1 correlate with the development of Kawasaki Disease and development of coronary artery aneurysms.

## Methods

FSTL-1 plasma levels were measured by ELISA in 48 patients with Kawasaki Disease at time of diagnosis (acute) and, 2 weeks, 6 weeks and 6 months following onset of disease. These were compared with plasma from 23 age-matched controls. In addition, FSTL-1 plasma levels were measured in 6 patients who developed coronary artery aneurysms. Data was analyzed using student’s t-test to evaluate for differences between FSTL-1 levels at different time points, and between patients who did or did not develop aneurysms.

## Results

FSTL-1 levels were significantly elevated in patients with acute Kawasaki Disease as compared to age-matched controls (mean 161.7 ng/ml vs. 121.3 ng/ml, p<0.001). The highest levels of FSTL-1 were observed in patients who developed coronary aneurysms (mean 225.5 ng/ml, p=0.005 compared to the acute time point ). FSTL-1 levels diminished during the subacute and convalescent phases of disease but still remained significantly elevated at 2 weeks and 6 weeks after disease onset. FSTL-1 levels returned to normal by 6 months.

## Conclusion

Plasma levels of FSTL-1 are elevated in acute Kawasaki Disease. Furthermore, FSTL-1 might be a valuable biomarker for predicting cardiac morbidity in this disease. Additionally, since FSTL-1 is produced by cardiac myocytes, these results suggest a possible role for FSTL-1 in the formation of coronary artery aneurysms.

## Disclosure

Mark Gorelik: None; David Wilson: University of Pittsburgh, 9; Stanford Shulman: None; Raphael Hirsch: University of Pittsburgh, 9.

